# Range accuracy in carbon ion treatment planning based on CT-calibration with real tissue samples

**DOI:** 10.1186/1748-717X-2-14

**Published:** 2007-03-23

**Authors:** Eike Rietzel, Dieter Schardt, Thomas Haberer

**Affiliations:** 1 Abteilung Biophysik, Gesellschaft für Schwerionenforschung, Planckstr. 1, 64291 Darmstadt, Germany.

## Abstract

**Background:**

The precision in carbon ion radiotherapy depends on the calibration of Hounsfield units (HU) as measured with computed tomography (CT) to water equivalence. This calibration can cause relevant differences between treatment planning and treatment delivery.

**Methods:**

Calibration data for several soft tissues were measured repeatedly to assess the accuracy of range calibration. Samples of fresh animal tissues including fat, brain, kidney, liver, and several muscle tissues were used. First, samples were CT scanned. Then carbon ion radiographic measurements were performed at several positions. Residual ranges behind the samples were compared to ranges in water.

**Results:**

Based on the measured data the accuracy of the current Hounsfield look-up table for range calibration of soft tissues is 0.2 ± 1.2%. Accuracy in range calibration of 1% corresponds to ~1 mm carbon ion range control in 10 cm water equivalent depth which is comparable to typical treatment depths for head and neck tumors.

**Conclusion:**

Carbon ion ranges can be controlled within ~1 mm in soft tissue for typical depths of head and neck treatments.

## Background

At the German carbon ion therapy facility Gesellschaft für Schwerionenforschung (GSI) more than 300 patients have been treated since 1997, primarily in the head and neck region [1,2]. The inverse depth dose profile, the so called Bragg curve, as well as the small lateral scattering of carbon ions allow to achieve good conformity between target volume and treated volume. The range of charged particles in tissue is determined by their primary energy as well as the tissue density distribution along the beam path. Therefore precise knowledge of ion stopping powers within the patient anatomy is essential for precise treatment planning.

At GSI, treatment planning is performed with the in-house treatment planning system Treatment Planning for Particles (TRiP) [3]. For optimization and dose calculations, patient CT data in Hounsfield units (HU) are transformed in a water-equivalent system. Already in 1979 Chen et al [4] as well as Mustafa and Jackson in 1983 [5] published the use of such range calibration tables and their significance for charged particle therapy. At GSI the transformation of CT HUs to water equivalence is based on a Hounsfield look-up table (HLUT) that was initially measured using tissue equivalent phantom materials as well as bovine and human bony tissues [6,7].

Methods to obtain and validate precise ratios between proton stopping powers and CT values have been systematically investigated at the Paul Scherrer Institut (PSI), Switzerland. Schneider et al. [8] reported a stoichiometric calibration of CT HUs to proton stopping powers. They conclude that tissue substitute calibrations should be used with caution. Their results were validated with proton radiographic measurements of a sheep head. The method of proton radiography as a tool for quality control in proton therapy had been previously published by Schneider and Pedroni [9]. Schaffner and Pedroni then reported the experimental verification of the relation between CT HUs and proton stopping powers for proton therapy treatment planning [10]. CT scans as well as proton radiographic measurements of several animal tissues and bone samples were performed. In conclusion, they expected that the range of protons in the human body can be controlled to better than ± 1.1% of the water equivalent range in soft tissue and ± 1.8% in bone, which translates into a range precision of about 1–3 mm in typical treatment situations. Recently Schneider et al reported the feasibility of optimizing the relation between CT-HUs and proton stopping powers patient specifically [11]. They acquired an in vivo proton radiograph of a dog patient treated for a nasal tumor. The HLUT was then optimized patient specifically and possible dosimetric consequences were assessed. The standard deviation between measured and calculated water equivalence was reduced from 7.9 to 6.7 mm when using the patient specifically optimized HLUT. Note that these standard deviations were derived from proton radiography and therefore correspond to uncertainties for penetrating the full extent of the dog head.

The most advanced method to obtain information on proton stopping powers in 3D is probably proton cone-beam computed tomography. The development of such a system for the acquisition of volumetric information on proton stopping powers was reported by Zygmanski et al from Massachusetts General Hospital [12]. Their feasibility study suggests that there may be some advantage in obtaining proton stopping powers directly with proton cone-beam CT.

The relation between carbon ion stopping powers and CT HUs has been extensively investigated at the National Institute of Radiological Sciences (NIRS) in Japan and at GSI. Matsufuji et al (NIRS) investigated the relationship between CT HU and electron density, scatter angle and nuclear reaction [13]. To assess conversion accuracy, they compared the method to determine HLUTs as reported by Chen et al [4] to that of PSI [8,10]. They concluded that Chen et al's method shows good agreement with real tissues in the lung to soft tissue HU region, whereas PSI's method retains good agreement over the entire HU range including bone. The difference between both methods reaches a maximum of 2.6% in the high HU region.

Kanematsu et al (NIRS) published a polybinary tissue model for radiotherapy treatment planning [14]. Body tissues are approximated by substitutes, namely water, air, ethanol, and potassium phosphate solution. Based on standard mixtures with known stopping powers, it is then possible to calibrate the relationship between CT HUs and carbon ion stopping powers by CT scanning of the samples only. The calibration method was successfully tested with biological materials.

**Figure 1:**
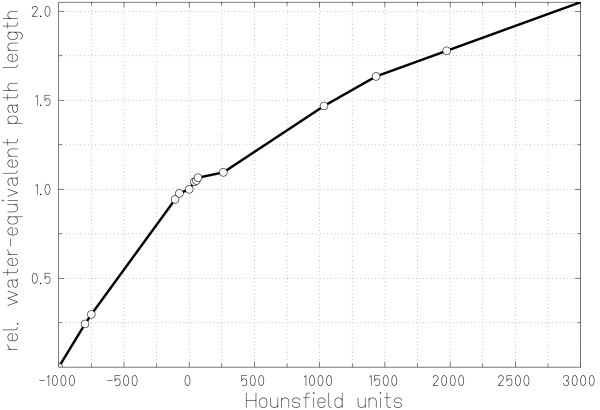
**Hounsfield look-up table for carbon ion treatment planning**. Measured data are plotted, connected by straight lines. Measurements were performed by Geiß et al [15] and Jäkel et al [7].

At GSI the initial HLUT calibration curve was determined by measuring CT HUs and integral carbon ion stopping powers of phantom materials [6]. Later, tissue equivalent materials as well as bovine and human bone tissues were used to improve the HLUT [7]. Inspired by the work at PSI, additional HLUT measurements were performed by Geiß et al using animal soft tissue samples [15]. Based on the measurements of Jäkel et al and Geiß et al, the HLUT was adapted, primarily in the soft tissue HU range. Figure 1 shows the current HLUT for carbon ion treatment planning at GSI.

In this work we present a summary of data for repeated measurements in the soft tissue HU region with different CT scanners to document the precision of the HLUT calibration curve. While quality assurance of the CT scanner calibration can routinely be performed with tissue equivalent materials as well as bone tissue samples once their integral stopping powers have been measured, this is not possible for soft tissues. For soft tissue samples CT HUs and integral stopping powers have to be measured on the same day. Measurements with soft tissues were repeated mainly for quality assurance and to assess accuracy of the HLUT in the soft tissue HU region. Some of the initial results have been reported previously [16-18].

## Methods

### Sample preparation

Fresh pig soft tissue samples were obtained directly from the butcher. These samples included brain, kidney, fat, liver, and various muscle tissues. Tissues were purified, for example fat was cut off muscle tissue and out of kidneys. Then each tissue was cut in blocks and wrapped in thin plastic foil (to avoid drying out) to fit into a PMMA box (inner dimensions 10 × 10 × 30 cm^3^, wall thickness 1 cm). The PMMA box was closed applying slight pressure. This was necessary to avoid shifting of the samples between CT scanning and carbon ion radiography. All measurements were performed within 12 hours after the pig was butchered.

### Computed Tomography

Two different CT scanner models were used for the HLUT measurement series in this study. Initially, a Siemens Somatom Plus 4 scanner (1), later a Siemens Somatom Volume Zoom scanner (2) was used. Image date were acquired according to a scan protocol for carbon ion therapy to ensure consistency between patient treatments and HLUT measurements. CT data were acquired in sequence scan mode slice by slice, reconstruction filter for the adult head (AH50), tube voltage of 120 kVp, and an integrated current of 420 mAs. CT voxel sizes were 1.29 × 1.29 × 1.00 mm^3 ^(1) and 1.38 × 1.38 × 1.00 mm^3 ^(2).

### Carbon ion radiography

**Figure 2:**
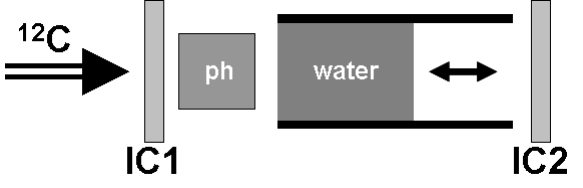
**Measurement setup for carbon ion radiography**. Residual ranges behind the phantom (ph) were measured by varying the thickness of the water absorber. Relative measurements were performed with two parallel plate ionization chambers (IC1, IC2).

Measurements of residual carbon ion ranges behind the samples were performed with a water absorber of variable thickness, a computer controlled water telescope. The measurement setup is shown in figure 2. Two parallel plate ionization chambers were used for relative measurements. The water absorber thickness was increased in steps of 200 μm to measure Bragg peak positions behind the samples. Different positions were irradiated using the magnetic raster scanning system [19]. This parallel scanning system allows to irradiate several measurement positions with carbon ion pencil beams without moving the tissue samples. Characteristics of the Gaussian shaped carbon ion pencil beam were energy 388 MeV/u (range in water 25.98 cm) and focus 2.3 mm at full width half maximum (FWHM).

For radiography measurements positions in homogeneous regions of the samples were selected. For example small inclusions of air within the tissue materials could not completely be excluded although special attention was paid to avoid air gaps during sample preparation. For paths in carbon ion beam direction (z-direction, orthogonal to slices), means and standard deviations of lines in the CT data were computed. These data were plotted similar to a projection to identify homogeneous tissue regions. Regions with low standard deviations per tissue sample were then selected for carbon ion radiography measurements.

**Figure 3:**
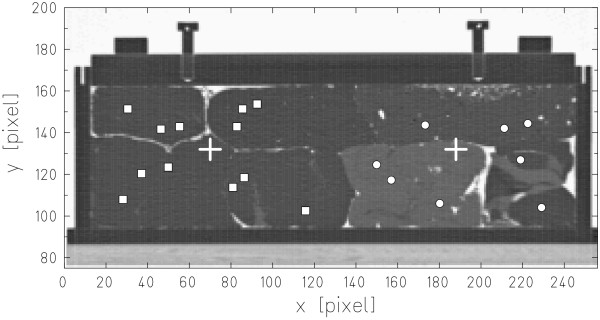
**Carbon ion radiography measurement positions**. Central slice of the PMMA phantom filled with different tissue samples (series 1). Carbon ion radiography measurements were performed at two different phantom positions, indicated by crosses. Positions selected for carbon ion radiographic measurements are indicated by squares and circles.

Figure 3 shows the central slice of the CT data for measurement series (1) including positions for carbon ion radiographic measurements. The PMMA box was positioned on the treatment couch according to the room laser system with CT slices orthogonal to the beam direction. To compare residual ranges behind soft tissue materials to range in water, additional measurements with the PMMA box filled with water were performed.

### Data analysis

Average CT HUs were calculated along the corresponding beam paths. Averaging was performed in a region over 5 × 5 pixels (beam FWHM 2.3 mm, pixel size ~1.3 mm) along the beam paths. Bragg peak positions were assessed by graphical inspection of the measured residual ranges. Because only relative differences between measurements were relevant for the analysis, carbon ion ranges were attributed to the maxima of the measured Bragg peaks. The water equivalent thickness of a specific tissue type is then given by

ρ=1+Δd
 MathType@MTEF@5@5@+=feaafiart1ev1aaatCvAUfKttLearuWrP9MDH5MBPbIqV92AaeXatLxBI9gBaebbnrfifHhDYfgasaacH8akY=wiFfYdH8Gipec8Eeeu0xXdbba9frFj0=OqFfea0dXdd9vqai=hGuQ8kuc9pgc9s8qqaq=dirpe0xb9q8qiLsFr0=vr0=vr0dc8meaabaqaciaacaGaaeqabaqabeGadaaakeaaiiGacqWFbpGCcqGH9aqpcqaIXaqmcqGHRaWkdaWcaaqaaiabfs5aebqaaiabdsgaKbaaaaa@3412@

with Δ shift of residual range behind the sample compared to water and d thickness of the sample. To assess the accuracy of the current HLUT, water equivalence for measured average HUs was calculated based on the current HLUT and compared to the measured water-equivalent path lengths (WEPL).

Small inclusions of air in the phantom as well as partial volume effects adjacent to the PMMA box's walls can affect the calculation of average HUs as well as residual range measurements. Voxels that clearly contained air, mainly between samples and PMMA box, were excluded for average HU calculation. Corresponding residual range measurements were consequently adjusted as well. Voxels containing air have a negligible stopping power in comparison to soft tissues and water. Therefore it is reasonable to simply subtract the distance of traversed air within the box from the residual range that was measured in the water telescope. This corresponds to virtually filling the air gaps with water.

Voxels with increased HUs adjacent to PMMA walls or obviously decreased HUs within or next to the sample tissues were excluded from average HU calculations only. This seems reasonable since radiographic measurements will not suffer from partial CT volume effects and voxels with slightly decreased HUs, e.g. from average 40 HU to local -100HU, are expected to consist of ~10% air (-1000 HU) and ~90% tissue (~40 HU).

## Results

**Figure 4:**
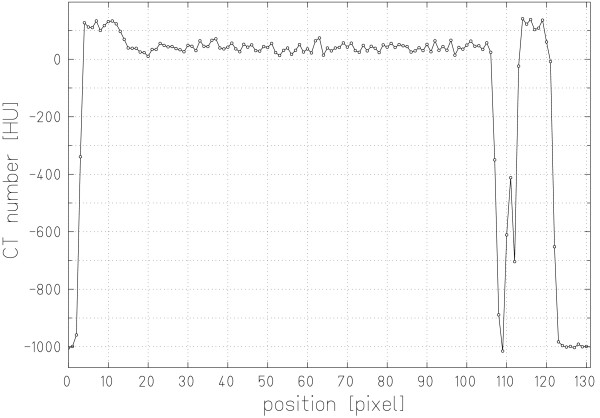
**Hounsfield units in brain tissue**. CT HUs along a radiography measurement path for brain tissue.

Figure 4 shows an example of HUs along the center of a beam path to illustrate our data analysis method. Note the air gap between the edge of the PMMA box and the brain tissue sample. For this example the average HU (40.9 ± 15.0) was calculated between the inner PMMA box walls excluding the voxels with obviously decreased HUs (2 voxels, HU above -500) and those mainly containing air (3 voxels, HU below -500). Including all voxels within the PMMA box, the average HU would be 5.7 ± 164.8. The measured residual range was adjusted as well. For each voxel that mainly consisted of air, the corresponding range in water was subtracted to calculate the WEPL (1.054). Another possibility to analyze the data would be to calculate the water-equivalent length of these 5 voxels according to our current HLUT. The relative difference in WEPL between the two methods is below 2% (1.037 vs. 1.054). Both values are within approximately ± 1% of our current HLUT. Because our measurements were performed to validate our HLUT we chose not to use the HLUT for data analysis.

**Figure 5:**
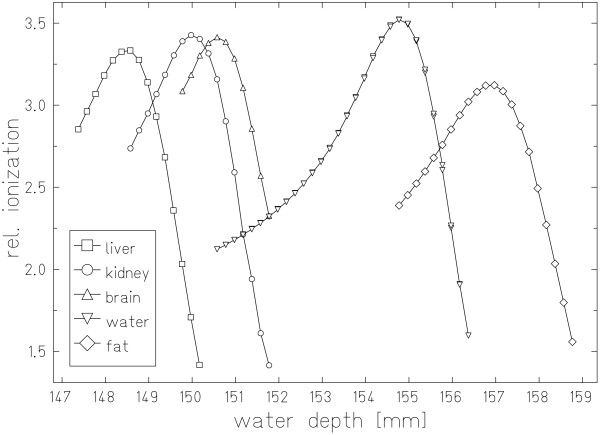
**Radiography measurement results**. Residual ranges measured with carbon ion radiography behind a PMMA box filled with different soft tissue samples and water.

Measured residual ranges behind the tissue sample as well as the water filled box are plotted in figure 5. Material specific shifts of the Bragg peaks according to the corresponding stopping powers are obvious. Different heights of the relative ionization signals result from small tissue inhomogeneities. In addition to range straggling these inhomogeneities lead to differences in ion ranges within a beam spot resulting in broadening of the depth dose profiles. This is most obvious for the Bragg peak measured behind the fat sample.

Results of different HLUT measurements are listed in table 1. In measurement series (1) 20 HLUT points and in series (2) 10 HLUT points were measured. Characteristics of relative WEPL differences compared to the current HLUT were (minimum, average ± standard deviation, maximum): (-1.1%, 0.6 ± 0.9%, 2.6%), (-2.6%, 0.6 ± 1.2%, 0.3%), and (-2.6%, 0.2 ± 1.2%, 2.6%) for measurement series (1), (2), and in total respectively. Relative differences were below -2% for 2, above 2% for 2, between -1% and -2% for 2, between 1% and 2% for 4, and within -1% and 1% for 20 measured HLUT points. Analysis of tissues involved in typical head and neck treatments, namely brain, fat, and neck, resulted in values of (-2.6%, 0.4 ± 1.4%, 2.6%).

**Table 1:** Comparison of measured and calculated Hounsfield look-up table points

	CT scanner 1			CT scanner 2		
	HU	WEPL	ΔWEPL	HU	WEPL	ΔWEPL

fat	-73.9 ± 20.8	0.978	-0.1 %	-97.9 ± 45.6	0.978	-2.6 %
	-72.7 ± 21.2	0.978	-0.0 %	-109.3 ± 43.6	0.960	-1.9 %
	-73.9 ± 27.6	0.980	-0.3 %	-102.6 ± 44.4	0.972	-2.5 %
brain	45.0 ± 17.4	1.044	-0.0 %	47.4 ± 16.0	1.042	0.3 %
	40.9 ± 15.0	1.054	-1.1 %	38.7 ± 18.6	1.041	-0.0 %
	44.0 ± 16.4	1.040	0.4 %			
kidney	53.1 ± 26.9	1.046	-0.0 %	49.0 ± 20.7	1.048	-0.2 %
	66.0 ± 15.8	1.041	2.1 %	54.0 ± 15.6	1.048	0.1 %
	57.5 ± 26.6	1.045	0.7 %			
liver	83.3 ± 20.5	1.059	0.8 %	75.5 ± 20.1	1.063	0.3 %
	79.7 ± 19.0	1.059	0.7 %	74.4 ± 27.6	1.064	0.2 %
	81.3 ± 13.7	1.061	0.6 %	72.9 ± 21.3	1.064	0.2 %
leg	65.5 ± 18.8	1.053	1.0 %			
	66.9 ± 23.6	1.072	-0.7 %			
	65.4 ± 19.6	1.049	1.3 %			
neck	66.0 ± 25.8	1.036	2.6 %			
	50.0 ± 47.5	1.043	0.4 %			
filet	73.3 ± 16.2	1.049	1.6 %			
	63.6 ± 25.5	1.049	1.1 %			
	69.5 ± 22.6	1.049	1.6 %			

HLUT measurements with two different CT scanners for fat, brain, kidney, and liver and additional measurements for various muscle tissues with CT scanner 1. Different samples of the same tissue type were used for measurements with CT scanner 1 and 2. To compare with measured data, relative differences in water equivalence in comparison to predictions based on the current HLUT are presented, positive numbers would result in over-ranges and negative values in under-ranges.

**Figure 6:**
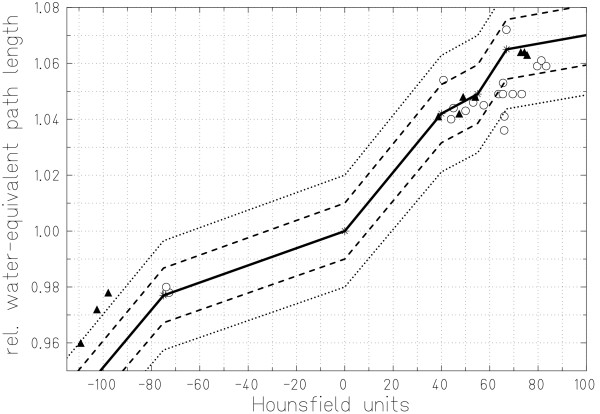
**Soft tissue region of the Hounsfield look-up table**. Soft tissue region of the current HLUT for treatment planning at GSI and measurements with two different CT scanners. Dashed and dotted lines represent the 1% and 2% confidence interval for WEPL calibration respectively.

Measured HLUT points as well as the current HLUT are plotted in figure 6. The dashed and dotted lines indicate the 1% and 2% confidence interval for WEPL calculation. Inspecting HLUT points per measurement series indicates that the HU calibration of the CT scanners might have been slightly different. Whereas points for all tissues besides fat of series (1) are predominantly shifted to slightly higher CT HUs, for series (2) the shift appears to be in the opposite direction for fat tissues only.

## Discussion

### Precision of measurements

Residual ranges were measured in 200 μm steps. The data in figure 5 demonstrate that determination of the Bragg peak positions is possible with at least the same precision. Radiographic measurements were performed for 10 cm of tissue. Uncertainties introduced by carbon ion radiography directly are therefore negligible. Only positioning errors of the samples could have an impact on radiography measurements because integral stopping powers would then be measured for the wrong beam paths. The phantom was aligned according to a laser system in the treatment room with a precision that can be expected to be better than 1 mm. By selecting the radiography positions based on HU averages and standard deviations along beam paths possible impacts of small positioning errors were further decreased.

One of the most critical tasks in charged particle radiotherapy is appropriate calibration of the CT scanner, concerning both, stability as well as reproducibility of absolute HUs. For slightly heterogeneous materials like soft tissue samples, it is not possible to differentiate between partial volume effects and tissue heterogeneities based on CT HUs. HU variations as denoted by the standard deviations along the radiography beam paths in table 1 can therefore not be analyzed further. The penetrated 10 cm of tissue correspond to 100 voxels. We expect this number of voxels to be sufficient for representative HU averages.

Systematic shifts between measured HU data and the current HLUT possibly occur for measurement series (1) in the region of 60 to 80 HU and and series (2) in the region of -100 to -110 HU. For series (1), the systematic shift is within the 1% HLUT confidence interval. For series (2), the shift in the fat tissue HU region is slightly outside of the 2% confidence interval. To possibly improve the HLUT calibration it might be necessary to generate a new calibration curve for scanner (2). However, another unceartainty can result from sample selection and preparation. The standard deviation for fat tissues was ~45 HU in measurement series (2) compared to ~20 HU in series (1). In combination with the decreased average HUs in series (2) for fat tissues, this indicates that most likely differences between the two samples were present that resulted in a relative WEPL difference of -2.6%.

The slightly higher standard deviations of HUs in comparison to the data reported by Jäkel et al [7] are attributed to the CT slice thickness of 1 mm in this study compared to 3 mm. We simulated 3 mm slice thickness by averaging 3 slices throughout the samples. For example the average HU for one of the brain tissue HLUT points then changes from 40.9 HU to 41.0 HU only, whereas the corresponding standard deviation decreases from 15.0 HU to 9.8 HU. For real measurements with 3 mm slice thickness further decrease of the standard deviations can be expected due to improved signal-to-noise ratios.

### Accuracy of patient treatments

In general our goal is to control the range of carbon ions within the patient to better than 1%. For typical patient treatments in the head and neck region water equivalent ranges to the target of approximately 10 cm can be expected. With range control of ~1% this results in a range uncertainty of ~1 mm. Schaffner et al (PSI) reported that they expect the range of protons to be controlled in soft tissue within 1.1% of the water equivalent range [10]. Our results are comparable. By repeated measurements we showed that on average the range of carbon ions in soft tissue can be reproduced with an accuracy of 0.2 ± 1.2%.

Another aspect of HLUT measurements are beam hardening effects as initially reported by Minohara et al [20]. They demonstrated the effect of different object sizes on the calibration of HUs to water equivalence. To date, only patients with tumors in the head and neck as well as in the pelvic region are treated at GSI [1,2]. We selected the dimensions of the PMMA box phantom to be comparable to typical head and neck dimensions because most of the tumors treated at GSI are located in this region, many of them directly abutting the brain stem. This ensures highest precision for treatments of head and neck tumors while slightly decreased range control might be expected for targets in the pelvic region.

## Conclusion

Calibration of CT HUs to water equivalence is critical to control the range of charged particles in the human body. With repeated measurements we found a precision for carbon ion range calibration in soft tissues of 0.2 ± 1.2%, and in soft tissues involved in typical head and neck treatment of 0.4% ± 1.4%. For soft tissues in typical patient treatments in the head and neck region this corresponds to a range uncertainty below 1 mm.

## Competing interests

ER is an employee of Siemens Medical Solutions, Particle Therapy. Measurements and analysis were performed while employed at GSI without other financial support.

## Authors' contributions

ER performed measurements, analyzed the data, and drafted the manuscript. DS and TH contributed to the study design, performed measurements and critically revised the manuscript. All authors read and approved the final manuscript.
